# Modelling multiple hospital outcomes: the impact of small area and primary care practice variation

**DOI:** 10.1186/1476-072X-5-50

**Published:** 2006-11-16

**Authors:** Peter Congdon

**Affiliations:** 1 Department of Geography, Queen Mary, University of London, Mile End Rd, London E1 4NS, UK.

## Abstract

**Background:**

Appropriate management of care – for example, avoiding unnecessary attendances at, or admissions to, hospital emergency units when they could be handled in primary care – is an important part of health strategy. However, some variations in these outcomes could be due to genuine variations in health need. This paper proposes a new method of explaining variations in hospital utilisation across small areas and the general practices (GPs) responsible for patient primary care. By controlling for the influence of true need on such variations, one may identify remaining sources of excess emergency attendances and admissions, both at area and practice level, that may be related to the quality, resourcing or organisation of care. The present paper accordingly develops a methodology that recognises the interplay between population mix factors (health need) and primary care factors (e.g. referral thresholds), that allows for unobserved influences on hospitalisation usage, and that also reflects interdependence between hospital outcomes. A case study considers relativities in attendance and admission rates at a North London hospital involving 149 small areas and 53 GP practices.

**Results:**

A fixed effects model shows variations in attendances and admissions are significantly related (positively) to area and practice need, and nursing home patients, and related (negatively) to primary care access and distance of patient homes from the hospital. Modelling the impact of known factors alone is not sufficient to produce a satisfactory fit to the observations, and random effects at area and practice level are needed to improve fit and account for overdispersion.

**Conclusion:**

The case study finds variation in attendance and admission rates across areas and practices after controlling for need, and remaining differences between practices may be attributable to referral behaviour unrelated to need, or to staffing, resourcing, and access issues. In managerial terms, the analysis points to the utility of formal statistical analysis of hospitalisation rates as a prelude to non-statistical investigation of primary care resourcing and organisation. For example, there may be implications for the location of staff involved in community management of chronic conditions; health managers may also investigate whether some practices have unusual populations (homeless, asylum seekers, students) that explain different hospital use patterns.

## Background

Of particular importance in strategic management of the primary-acute care interface is identification of the sources of variation in hospital referrals and attendances, and detecting whether particular small areas and GP practices have above average emergency attendance and admission rates, especially where such variations are not related to acknowledged sources of health need. Containment of unplanned emergency admissions to, and attendances at, hospital emergency units is a major element in strategic management of the health demand generated by long term chronic illness, as hospital based (acute) care is relatively costly. If excess referral rates are indicated for particular practices this may have implications for clinical and organisational effectiveness in primary care – see, for example [1]. However, a statistical analysis of the kind undertaken here can only identify referral variations per se, and not necessarily make inferences on effectiveness.

In the recent past, the emphasis was on reducing avoidable emergency attendances or admissions, for conditions such as asthma, urinary tract infections and chronic obstructive pulmonary disease that are treatable in a primary setting given timely and appropriate care [2]. In the last few years, the UK's National Health Service (NHS) strategy has switched to containment of emergency care demand in general, with the government seeking to reduce unplanned emergency admissions to hospital for long-term chronic conditions [3,4], for example, by greater use of community matrons [5] assigned to patients with high intensity health needs and by encouraging primary and community care options for such patients wherever possible. Such initiatives are sometimes grouped as constituting the "chronic care model" [6]. Their cost effectiveness has been demonstrated for appropriately selected conditions [7] – that is, care is cheaper without any deterioration in clinically defined measures of patient health state [8].

GP practices have a major role in managing the demand generated by long term conditions but may vary in their referral rates for avoidable emergencies [9] or have access arrangements that leave patients with little choice but to resort to hospital emergency units [10,11]. Some studies have concluded that there are significant variations in practice referral behaviour [12,13], and the UK government has identified wide variations [4] in rates of unplanned emergency admissions between health areas. Variations in referrals unrelated to need may in part be related to resourcing mechanisms that do not sufficiently compensate for variations in ill health between population subgroups [14]; spatial access issues are also important [15]. Improved resourcing of, and access to, primary care in deprived areas means primary and community care teams will be better able to address patient morbidity in community settings, and patients will be less likely to attend hospitals for minor emergencies.

The present paper develops methods that recognises the interplay between population mix factors (e.g. social composition, health need and genuine morbidity differences), access, and primary care factors, that allows for unobserved influences on hospitalisation usage, and that also reflects interdependence between responses. Tools such as these may assist health care agencies to take actions to improve equity in access to primary care, to target chronic care model initiatives and to improve the match of resources to need, with the aim of reducing unplanned emergency admissions or attendances at hospitals.

### Contextual Setting

In the UK health system, patients may choose their GP practice and there are no geographic constraints on their choice, so patients resident in small areas i = 1,...,I are typically affiliated to a range of practices (j = 1,..,J), though in practice geographic access to the GP surgery strongly affects choice of practice. This paper proposes a modelling strategy for multiple health outcomes (e.g. admissions, attendances) where "outcomes" is used in a broad statistical sense as a response variable rather than the health care sense. The strategy allows for the impact on referral rates of known factors such as area social structure (e.g. area deprivation), and differences in geographic accessibility to both primary care and to hospitals with emergency units. It also allows for unknown influences taken to vary randomly over areas and practices. This involves a cell decomposition approach whereby area populations (and the hospitalization or other health care events they generate) are disaggregated according to the primary care practices they are affiliated to. So variations for multiple outcomes k = 1,..,K are considered for population totals P_ij _classified both by area i and GP practice j.

Since in fact many cells in the I × J cross-classification of patients by area and practice are empty or contain very few patients (e.g. because practice j is too geographically remote from area i to attract any patients from it), the method of analysis in this paper uses a subset of the IJ possible area-practice intersections. This involves including area-practice cells which account for the great majority of a practice's population, but excluding those that may account for just a few patients. So the observations are totals Y_kh _by outcome k and cell h = 1,..,H, where H is less than IJ, and where i_h _and j_h _are the area and practice corresponding to cell h. Here K = 2, and attendances and admissions Y_kh _(k = 1 for attendances, k = 2 for admissions) are considered in relation to expected cases in the population Pihjh
 MathType@MTEF@5@5@+=feaafiart1ev1aaatCvAUfKttLearuWrP9MDH5MBPbIqV92AaeXatLxBI9gBaebbnrfifHhDYfgasaacH8akY=wiFfYdH8Gipec8Eeeu0xXdbba9frFj0=OqFfea0dXdd9vqai=hGuQ8kuc9pgc9s8qqaq=dirpe0xb9q8qiLsFr0=vr0=vr0dc8meaabaqaciaacaGaaeqabaqabeGadaaakeaacqqGqbaudaWgaaWcbaGaeeyAaK2aaSbaaWqaaiabbIgaObqabaWccqqGQbGAdaWgaaadbaGaeeiAaGgabeaaaSqabaaaaa@33D1@ living in area i_h _and affiliated to practice j_h_.

Various factors influencing attendance and admission rate variations between areas may be unobserved. The modelling strategy therefore includes an allowance for spatially correlated but unobserved risk factors for areas; these are modelled by an intrinsic conditional autoregressive or ICAR prior [16]. Similarly a practice level random effect summarises influences on practice referral rates that cannot be accounted for by known measures of practice health needs. Different approaches are possible to model correlated need and behaviour: for example, one may use a K-variate ICAR prior for area spatial effects combined with K-variate practice errors. Another option is a common factor approach which is less heavily parameterised (especially as K increases), and will be effective when there are high correlations in area and practice relativities over the outcomes.

The method extends the approach of Congdon and Best [17] to multivariate outcomes. It is adapted to a situation where multiple outcomes (e.g. attendances and admissions) are interrelated in terms of morbidity influences (variations in health need due to known and unknown factors) and also behavioural influences (e.g. referral thresholds of GP practices).

The methods described in the paper adopt a fully Bayesian strategy. This involves specification of prior densities on parameters and updating such densities (to provide posterior parameter densities) via the likelihood of the observed data. In estimating the models, iterative Monte Carlo Markov Chain (MCMC) techniques [18] are used, as implemented in the WINBUGS program [19].

### Case Study

A case study analysis uses data on 20200 attendances and 5970 emergency admissions at Oldchurch Hospital in North East London during April 1 – September 30^th ^2003, and focuses on attendances at, and admissions to, this hospital by residents of the outer London borough of Havering, within which the hospital is located. The majority (93.7%) of the 239 thousand residents of Havering (according to the NHS patent register) are affiliated to one of J = 53 GP practices sited within Havering; around 15000 are affiliated to practices outside Havering.

The data sources are standard NHS patient recording systems. The attendances data are based on a patient administration system maintained by the Barking, Havering and Redbridge Trust (BHRT), while the admissions data are from the centrally collated NHS Health Episode Statistics, widely used (e.g. in monitoring government health targets) to analyse morbidity, access and utilisation in the English health care system. Oldchurch accounted for around 80% (5970/7510) of emergency admissions by Havering residents in the period. It is not possible to ascertain how many attendances there are by Havering residents to all hospitals in the surrounding region, since attendance data are not collated centrally, and individual trusts have their own systems (or may not have attendance recording systems at all); attendances by Havering residents at Oldchurch greatly exceed those at King George, the other BHRT hospital with an emergency unit. Patient populations by age and sex are obtained from the National Health Service Central Register [20] which holds demographic details for patients registered with General Practitioners in different health areas – currently such areas are known as Primary Care Trusts (PCTs) and Havering PCT is coterminous with the London Borough of Havering. Census data on long term illness are from the 2001 Census, as discussed below.

**Figure 1:**
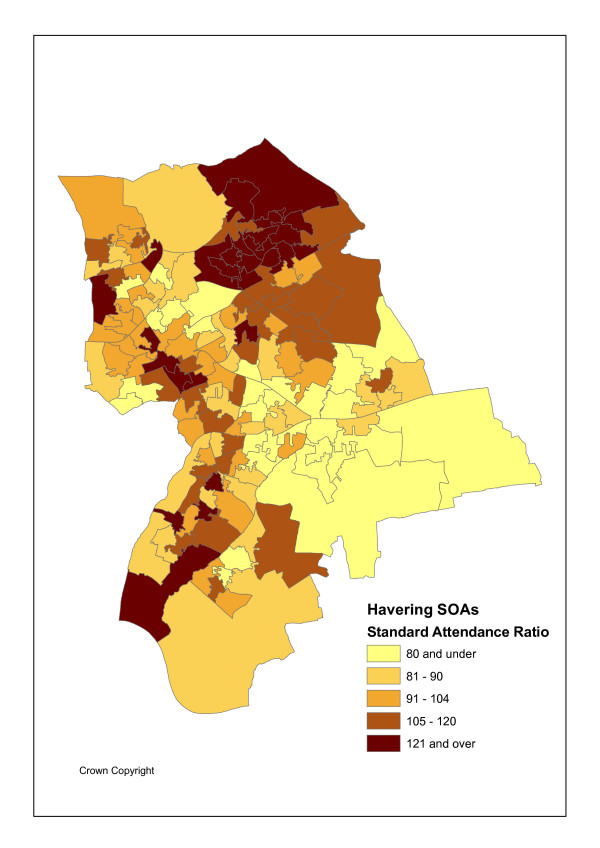
SARs for Emergency Attendances.

**Figure 2:**
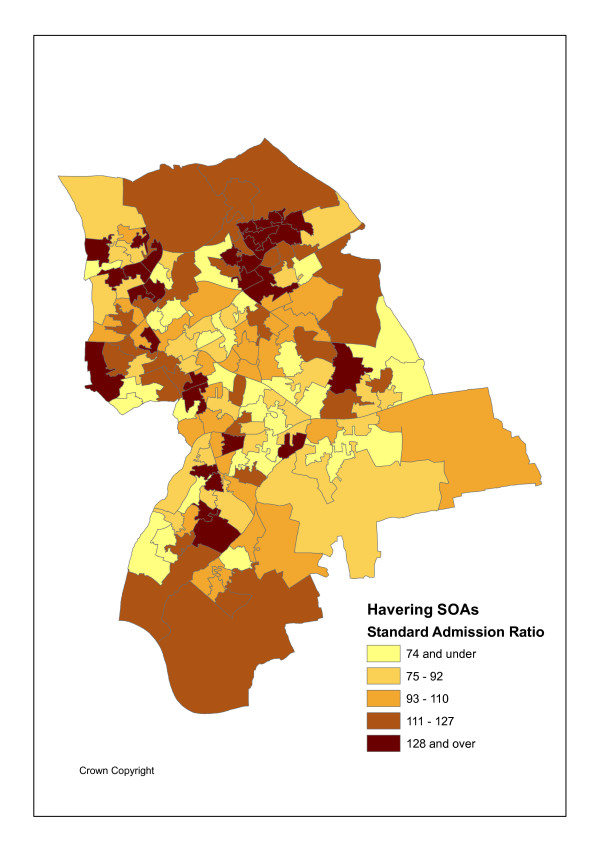
SARs for Emergency Admissions.

**Figure 3:**
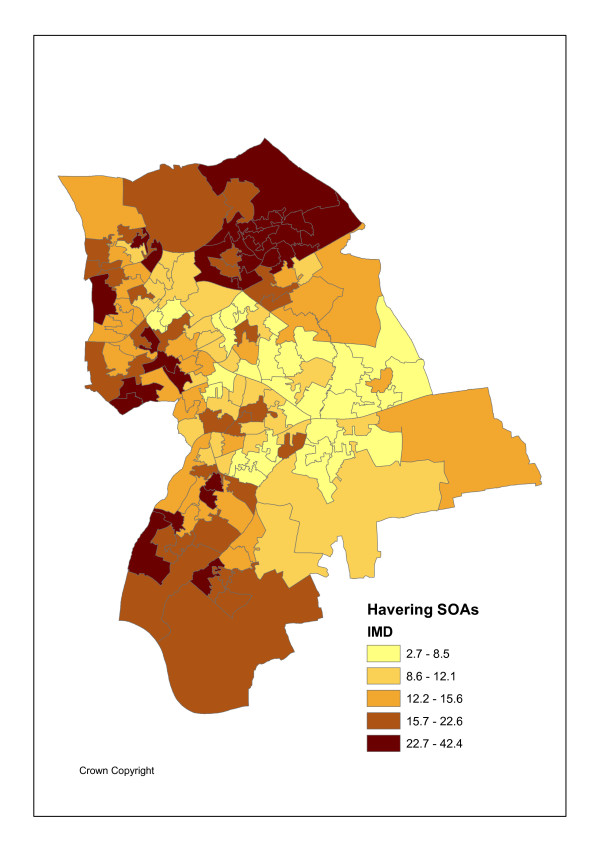
IMD2004 Multiple Deprivation Score.

The geographical unit of analysis is the recently introduced Super Output Area (abbreviated as SOA), designed by the UK Office of National Statistics Census Unit SOAs to avoid the problems caused by the inconsistent and unstable electoral ward geography and improve statistical comparison as they are of consistent size [21]. Specifically lower level SOAs are used here. There are I = 149 such SOAs in Havering with an average population P_i _of 1500. Figures 1 and 2 show the variation in maximum likelihood SOA attendance and admission ratios, namely standardised activity ratios (SARs) of observed to expected events. Figure 3 shows SOA indicators for deprivation rank (within Havering), as measured by Index of Multiple Deprivation (IMD) scores for 2004 [22]. These maps use quintile breaks.

For the regression modelling of area and practice effects, the threshold approach mentioned above for including area-practice cells was applied with respect to SOA populations. If a GP practice accounted for more than 1% of an area's population the corresponding area-practice cell was included in the study. Under this definition, 97.7% of Havering's population who are affiliated to a Havering practice are included in the analysis, and there are H = 1620 cells.

Indirect standardisation is chosen in Figures 1 and 2 and in the regression analysis below because it is appropriate in the event of the small attendance/admission totals (including zeroes), and also often small population denominators. The regression analysis is at the area-practice population cell level and direct standardisation would involve cell level rates disaggregated by age and sex, obtained by dividing cell level events in each of 38 age-sex categories by cell level populations in each category. There are over 1600 cells in a population of around 220 thousand, so the population denominators involved are often small and the rates obtained have high sampling variability.

To quote the Clinical Indicators Support Team of NHS Scotland [23], "direct standardisation is inadvisable if the number of cases in any of the cells of the cross-classification of the variables used to standardise is small. Thus if one is standardising for age, sex and deprivation and there is a possibility of very low numbers in any combination of age, sex and deprivation categories, direct standardisation should be avoided. If there is a possibility that there are no cases in any of the cells of classification (zero cells) then direct standardisation is entirely ruled out. Indirect standardisation is highly robust in the context of small cell numbers". A further feature of indirect standardisation reported by Kendrick & McCloed [24] "is that..it is difficult to envisage a more robust technique for case mix adjustment. Not only does it adjust for the effects of individual variables, such as age, on outcome, it automatically adjusts for any interactions resulting from particular combinations of case mix variables. Indirect standardisation literally adjusts for the effects of all combinations of the casemix variables. ... indirect standardisation produces exactly the same results (is logically equivalent to) standardisation based on a fully saturated logistic regression model".

In the analysis below the implementation of indirect standardisation can be represented generically by considering the outcome Y_s _for stratum s (e.g. area, practice or cell) as a Poisson variable with mean μ_s _= E_s_ρ_s_, where E_s _is the expected outcome total or "exposure" for a Poisson response; for a fuller discussion of this method see, for example, [25] and [26]. Written out as a log link regression one has log(μ_s_) = log(E_s_) + log(ρ_s_) and since the implicit coefficient on log(E_s_) is 1 the E_s _term is sometimes called an offset [27].

In the present application the expected events E_s _are based on internal standardisation using data from the GP Population Register on the age-sex structure of the population at risk, and with the standard rates based on the entire Havering population included in the analysis. When expected outcomes are calculated using internal standardisation in this way, the ρ_s _can be interpreted as relative risks with average 1.

### Patterns in Maximum Likelihood Activity Ratios

There are strong associations between standardised attendance and admission rates and social deprivation (IMD scores for 2004) in the case study population [22]. Such scores can also be calculated for GP practices by averaging over the scores for their patient places of residence. Table 1 shows the highest all ages attendance and admission ratios are in deprived SOAs and practices. There are positive correlations (of 0.65 and 0.72 respectively) between maximum likelihood SOA attendance and admission ratios (ratios of observed to expected events, Y_ik_/E_ik_) and the IMD scores for such SOAs.

**Table 1:** Maximum Likelihood Activity Ratios (Admissions & Attendances) by Deprivation Category, SOAs and GP Practices

**SOA IMD Score**	**Standard Attendance Ratio (× 100)**	**Standard Admission Ratio (× 100)**	**Practice IMD Score**	**Standard Attendance Ratio (× 100)**	**Standard Admission Ratio (× 100)**
Under 8.5	81	80	Under 10.5	72	84
8.5–12.0	93	87	10.5–14.3	86	92
12.0–15.0	104	101	14.3–16.5	124	108
15.0–22.5	115	104	16.5–18	98	109
Over 22.5	121	125	Over 18	120	132

One may apply significance tests (from a classical rather than Bayesian perspective) to the practice or area SARs or functions of them to assess possible outliers. Suppose a high referring practice is defined as one with a SAR exceeding 150 and with the 2.5% confidence point exceeding 100. Using the procedure proposed in [28], five practices (11,19,35,42,49) have standard attendance ratios and 95% intervals on them fulfilling this criterion, and four practices (11,35,36,50) have 95% standard admission ratios fulfilling the criterion. Practices 11 and 35 are outliers for both attendances and admissions.

However, such fixed effects maximum likelihood estimates (and tests based on them) are often based on relatively small event numbers and also do not take account of spatial correlation in adjacent area rates or of the potential for pooling inferential strength by considering the underlying population density of rates over areas, practices, or cells. As Leyland and Davies [29] mention, "a map displaying [maximum likelihood fixed effect] SMRs tends to be dominated by areas with small populations since small changes to the observed number of deaths will result in large changes to the SMR". The approach of the present paper is based on the principle of pooling strength over related units (such as areas, schools, hospitals or GP practices) by modelling the density underlying the population of effects, after controlling for known influences on such effects. This approach is well known in applications in disease mapping and meta-analysis – for a fuller discussion, see for example [30] and [31].

Random effects may also be needed to model excess heterogeneity or overdispersion [32] relative to that expected under the Poisson model – this is measured by comparing the scaled deviance [33] with the number of observations. As noted in [34], "One of the most common reason for data being over-dispersed is that experimental conditions are not perfectly under control, and thus the unknown parameters vary not only with measured covariates but with latent and uncontrolled factors." Nevertheless a fixed effects model based on measured covariates only is included in the case study as a baseline model.

## Methods

### Models for Geographical and Practice Variations: Fixed Effects Model based on Known Influences

The analysis of this paper is predicated on looking at both area (SOA) and practice effects simultaneously via a cell level analysis. This is argued to be relevant for a *crossed *analysis of hospital outcomes and provides control for the catchment area effects on a GP practice's workload (e.g. a practice has a high referral rate because it catchment area has a population with high morbidity). Conversely, a cell analysis also controls for the possibility that area effects are distorted by possibly unusual practice referral rates (e.g. when a GP practice in a low morbidity area has an unusually high referral propensity, due possibly to varying referral thresholds). By comparison, analysis at practice level alone, or at SOA level alone is partial, and neglects area-practice interactions. (Analogous problems of ecological or atomistic distortions occur when a single rather than multilevel analysis is performed with *nested *as opposed to crossed data). Therefore an appropriate regression analysis is at the area-practice cell level.

The model used here for event k totals from cell h (admissions Y_1h _and attendances Y_2h_) takes them to result from risks of the event in the area i_h _and in the GP practice j_h _that define cell h. Assuming Poisson variation in the counts

Y_kh _~ Po(E_kh _ρ_kh_),     k = 1, ... K     (Equation 1)

where E_kh _are expected admissions/attendances based on applying the study area wide (i.e. Havering) age-sex rates to the cell populations and ρ_kh _is the relative event rate in cell h (with mean 1 if internal standardisation is used). Define

log(ρ_kh_) = α_k _+ η_kh _    (Equation 2)

where α_k _is a constant term.

One possible approach to analyzing variations in referral rates over cells is to use Poisson regression (because the responses are counts) but without allowing for unobserved area or practice influences. Thus the regression would use only known attributes of areas, practices or cells in a *fixed effects *model.

The first such class of indicators are needs scores for practices and areas. The models below are framed in terms of composite area and practice health need scores. There are several potential indicators of need at area and/or practice level such as the IMD2004 total deprivation score, and more specific domain scores: namely the income domain score (the proportion of the population experiencing income deprivation in an area), and the "health, deprivation and disability" (HDD) domain. Another possibility is the 2001 Census standard illness ratio [35]. Such scores are calculated for GP practices by averaging over the scores for their patient places of residence. However, correlations between these indicators are high within the SOAs and within practices (see Table 2). Including (say) as independent variables both the SOA income scores and SOA health domain scores together with the practice level income and health domain scores might seem to be representing subtly different aspects of need, but would be at the expense or distorting the regression results.

**Table 2:** Correlations of needs scores* over practices & SOAs

(a) Correlations over practices			
	HDD-DOM2004	SIR2001	IMD2004
INCDOM2004	0.952	0.979	0.934
HDD-DOM2004		0.979	0.966
SIR2001			0.954
(b) Correlations over SOAs			
	HDD-DOM2004	SIR2001	IMD2004
INCDOM2004	0.875	0.897	0.951
HDD-DOM2004		0.903	0.915
SIR2001			0.890

* ABBREVIATIONS: HDD-DOM = Health Deprivation & Disability Domain; INCDOM = Income domain; SIR = standard illness ratio (2001 Census)

For simplicity therefore, the model specification is framed in terms of a single needs score for SOAs, and a single needs score for practices. These are denoted AHN_i _and PHN_j _respectively and have mean 0 and variance 1. They are based on maximum likelihood factor analysis (in SPSS) of the four indices in Table 2. The eigenvalues from the correlation matrix show overwhelming support for there being only a single underlying need score for areas and for practices.

Other known influences on hospital utilisation at area level include the Euclidean distance to the case study hospital (R_i_), and access to primary care A_i_. The access score A_i _is based on the Euclidean distances d_ij _between SOAs and the GP practices j = 1,..,J (J = 53) that are based in Havering. This score takes account of the number M_j _of GPs in each GP practice. Thus

Ai=∑j=1JMjf(dij)     (Equation 3)
 MathType@MTEF@5@5@+=feaafiart1ev1aaatCvAUfKttLearuWrP9MDH5MBPbIqV92AaeXatLxBI9gBaebbnrfifHhDYfgasaacH8akY=wiFfYdH8Gipec8Eeeu0xXdbba9frFj0=OqFfea0dXdd9vqai=hGuQ8kuc9pgc9s8qqaq=dirpe0xb9q8qiLsFr0=vr0=vr0dc8meaabaqaciaacaGaaeqabaqabeGadaaakeaacqqGbbqqdaWgaaWcbaGaeeyAaKgabeaakiabg2da9maaqahabaGaeeyta00aaSbaaSqaaiabbQgaQbqabaGccqqGMbGzcqGGOaakcqqGKbazdaWgaaWcbaGaeeyAaKMaeeOAaOgabeaakiabcMcaPaWcbaGaeeOAaOMaeyypa0JaeGymaedabaGaeeOsaOeaniabggHiLdGccaWLjaGaaCzcamaabmaabaGaeeyrauKaeeyCaeNaeeyDauNaeeyyaeMaeeiDaqNaeeyAaKMaee4Ba8MaeeOBa4MaeeiiaaIaeG4mamdacaGLOaGaayzkaaaaaa@503E@

where f(d) is a declining function of the distance d_ij _between the population centroid of area i and the location of the surgery of practice j. Where a practice has a branch surgery (5 out of 53 practices) the distance calculation is to the branch surgery. Here exponential decay is assumed, in line for instance with the health resource analysis by [36], such that

f(d_ij_) = exp(-bd_ij_)     b>0     (Equation 4)

It is expected that an analysis based on travel times as opposed to Euclidean distance would give similar results. Use of travel times is problematic as visitors to GP surgeries may use one of several alternative modes, and additionally Euclidean distances tend to be strongly correlated with travel times [37].

It is also necessary to take account of the impact on cell-level attendance and admission rates of variations in primary care referral behaviours. As well as the practice's overall population morbidity level represented in the composite need score PHN_j_, the proportion NH_j _of the elderly (over 75) practice population that are living in nursing and residential homes may be relevant. These homes contain high proportions of frail elderly and are subject to high hospitalisation rates.

Other known practice predictors that may influence referrals were included in exploratory work (whether the practice is single handed and list size to GP ratio) but were not significant. These are also not indicators of health need per se, but of practice organisation. The goal here is to control for the impact of health need on practice behaviour and assess residuals as indicative of variations in referral or attendance unrelated to need. It is important to contextualise GP practice variations for known population casemix factors as relatively good or bad 'performance' indicators for (say) health providers or schools may result in part from the social structure and health need of their population [38].

Then a *fixed effects *approach using known influences only will be based on SOA need, primary care access, and distance to hospital, and practice need and nursing home patients. So for cell h and outcome k the fixed effects regression is

η_kh _= γ_k1_AHN_i _+ γ_k2_A_i _+ γ_k3_R_i _+ β_k1_PHN_j _+ β_k2_NH_j_.     (Equation 5)

This model can be estimated by classical techniques (e.g. in STATA or R), or by Bayesian techniques. Except for small samples, classical and Bayesian estimates of such a model are usually very similar.

### Random Effects Options

Even after accounting for known impacts on need and access to care, there are likely to remain unknown factors influencing hospital usage outcomes. Consider unknown area and practice influences on hospitalisation rates. The contribution of the present paper lies particularly in specifying *random effects *for such unknown factors in analysis of attendances Y_1h _and admissions Y_2h _in cell h, that is in SOA i_h _and practice j_h_, while taking account of relevant known area and practice influences. While classical estimation of random effects models has progressed in recent years, the view taken in the current paper is that Bayesian estimation considerably facilitates the estimation of relatively complex random effects models [39].

Unknown influences may be correlated over areas, outcomes or both. For example, neighbouring areas may experience similar impacts on morbidity (and hence hospitalisation) from unknown environmental factors. These effects are also likely to be correlated across outcomes, implying the need for a multivariate spatial error. For instance, densely populated areas close to major traffic routes may show both high attendance and admission rates for conditions related to air and traffic pollution. For practices, unobserved variations in practice referral behaviour are likely to be correlated over outcomes.

Consider first a K-dimensional error structure for both area and practice random effects. Let s_ki _denote spatially correlated influences on morbidity for outcome k and areas i. For modelling correlation between s_ki _and s_mi _(k ≠ m), Gelfand and Vounatsou [40] propose a multivariate conditional autoregressive (MCAR) prior. Let S_i _= (s_1i_,...s_Ki_) be the multidimensional error for the i^th ^area. Then under the MCAR prior, S_i _has a conditional prior density

Si|S[i],ρ,Σs)∼NK(ρ∑j∼iWijSj,Σs/∑jcij)     (Equation 6)
 MathType@MTEF@5@5@+=feaafiart1ev1aaatCvAUfKttLearuWrP9MDH5MBPbIqV92AaeXatLxBI9gBaebbnrfifHhDYfgasaacH8akY=wiFfYdH8Gipec8Eeeu0xXdbba9frFj0=OqFfea0dXdd9vqai=hGuQ8kuc9pgc9s8qqaq=dirpe0xb9q8qiLsFr0=vr0=vr0dc8meaabaqaciaacaGaaeqabaqabeGadaaakeaacqqGtbWudaWgaaWcbaGaeeyAaKgabeaakiabcYha8jabbofatnaaBaaaleaacqGGBbWwcqqGPbqAcqGGDbqxaeqaaOGaeiilaWIaeqyWdiNaeiilaWIaeu4Odm1aaSbaaSqaaiabbohaZbqabaGccqGGPaqkcqWI8iIocqqGobGtdaWgaaWcbaGaee4saSeabeaakiabcIcaOiabeg8aYnaaqafabaGaee4vaC1aaSbaaSqaaiabbMgaPjabbQgaQbqabaGccqqGtbWudaWgaaWcbaGaeeOAaOgabeaaaeaacqqGQbGAcqWI8iIocqqGPbqAaeqaniabggHiLdGccqGGSaalcqqHJoWudaWgaaWcbaGaee4Camhabeaakiabc+caVmaaqafabaGaee4yam2aaSbaaSqaaiabbMgaPjabbQgaQbqabaaabaGaeeOAaOgabeqdcqGHris5aOGaeiykaKIaaCzcaiaaxMaadaqadaqaaiabbweafjabbghaXjabbwha1jabbggaHjabbsha0jabbMgaPjabb+gaVjabb6gaUjabbccaGiabiAda2aGaayjkaiaawMcaaaaa@6D71@

where N_K _denotes a K dimensional normal density, S_[i] _denotes spatial effects other than those in area i, ρ∈[0,1] is a scalar, Σ_s _is a K × K covariance matrix, and W_ij _= w_ij_I_K × K _is K × K with w_ij _denoting row standardized spatial interactions, namely wij=cij/∑jcij
 MathType@MTEF@5@5@+=feaafiart1ev1aaatCvAUfKttLearuWrP9MDH5MBPbIqV92AaeXatLxBI9gBaebbnrfifHhDYfgasaacH8akY=wiFfYdH8Gipec8Eeeu0xXdbba9frFj0=OqFfea0dXdd9vqai=hGuQ8kuc9pgc9s8qqaq=dirpe0xb9q8qiLsFr0=vr0=vr0dc8meaabaqaciaacaGaaeqabaqabeGadaaakeaacqqG3bWDdaWgaaWcbaGaeeyAaKMaeeOAaOgabeaakiabg2da9iabbogaJnaaBaaaleaacqqGPbqAcqqGQbGAaeqaaOGaei4la8YaaabuaeaacqqGJbWydaWgaaWcbaGaeeyAaKMaeeOAaOgabeaaaeaacqqGQbGAaeqaniabggHiLdaaaa@3ECE@. If the w_ij _are based on contiguity (that is, c_ij _= 1 if SOAs i and j are adjacent, and c_ij _= 0 otherwise), then w_ij _= 1/L_i _if areas i and j are adjacent, with L_i _being the number of neighbours of area i. The introduction of ρ ensures the corresponding joint prior is proper, with nonsingular covariance matrix. If ρ is set to 1, as in

Si|S[i],ρ,Σs∼NK(∑j∼iWijSj,Σs/∑jcij)     (Equation 7)
 MathType@MTEF@5@5@+=feaafiart1ev1aaatCvAUfKttLearuWrP9MDH5MBPbIqV92AaeXatLxBI9gBaebbnrfifHhDYfgasaacH8akY=wiFfYdH8Gipec8Eeeu0xXdbba9frFj0=OqFfea0dXdd9vqai=hGuQ8kuc9pgc9s8qqaq=dirpe0xb9q8qiLsFr0=vr0=vr0dc8meaabaqaciaacaGaaeqabaqabeGadaaakeaacqqGtbWudaWgaaWcbaGaeeyAaKgabeaakiabcYha8jabbofatnaaBaaaleaacqGGBbWwcqqGPbqAcqGGDbqxaeqaaOGaeiilaWIaeqyWdiNaeiilaWIaeu4Odm1aaSbaaSqaaiabbohaZbqabaGccqWI8iIocqqGobGtdaWgaaWcbaGaee4saSeabeaakiabcIcaOmaaqafabaGaee4vaC1aaSbaaSqaaiabbMgaPjabbQgaQbqabaaabaGaeeOAaOMaeSipIOJaeeyAaKgabeqdcqGHris5aOGaee4uam1aaSbaaSqaaiabbQgaQbqabaGccqGGSaalcqqHJoWudaWgaaWcbaGaee4Camhabeaakiabc+caVmaaqafabaGaee4yam2aaSbaaSqaaiabbMgaPjabbQgaQbqabaaabaGaeeOAaOgabeqdcqGHris5aOGaeiykaKIaaCzcaiaaxMaadaqadaqaaiabbweafjabbghaXjabbwha1jabbggaHjabbsha0jabbMgaPjabb+gaVjabb6gaUjabbccaGiabiEda3aGaayjkaiaawMcaaaaa@6AD9@

then a propriety issue occurs, though identifiability may be achieved by centering each of the K sets of effects at each MCMC iteration. This approach is used in WINBUGS and in the analyses reported here.

Then the model for the area component of η_kh _involves area i_h _that together with practice j_h _defines cell h. So for i = i_h_

η^(a)^_ki _= γ_k1_AHN_i _+ γ_k2_A_i _+ γ_k3_R_i _+ s_ki _    k = 1,...,K     (Equation 8)

where the remaining parameters are assigned fixed effect priors. Estimates of the log relative risk of event k for area i may be obtained by monitoring the quantities η^(a)^_ki _centred round their average over all SOAs. Posterior means of the exponentials of centred η^(a)^_ki _can be obtained to provide estimates μ_ki _of relative risk in area i.

There will also be many unobserved influences on primary care effectiveness that are here initially summarised in a practice level random effect for each outcome e_kj_. Then a K-variate error model for the GP practice component of η_kh _includes the composite practice health need score and nursing home effects, and errors e_kj_. So with j = j_h_

η^(p)^_kj _= β_k1_PHN_j _+ β_k2_NH_j _+ e_kj _    (Equation 9)

where e_j _= (e_1j_, e_2j_) are bivariate normal random effects with mean zero and covariance Σ_e_. So with K = 2

(e_1j_, e_2j_) ~ N_2_(0, Σ_e_),     j = 1,..., j.     (Equation 10)

Posterior means of the exponentials of centred η^(p)^_kj _can be obtained to provide estimates of relative risk ν_kj _in practice j.

If a model based on practice and area predictors and random effects leaves substantial unexplained variation (i.e. produces a model from which predictions do not match the observations), it may be necessary to add predictors and/or random effects at cell level. For example, multivariate cell level random effects u_h _= (u_1h_, u_2h_,..u_Kh_), h = 1,...H, may be needed to represent remaining heterogeneity, as in

log⁡(ρkh)=αk+ηkih(a)+ηkjh(p)+ukh     (Equation 11)
 MathType@MTEF@5@5@+=feaafiart1ev1aaatCvAUfKttLearuWrP9MDH5MBPbIqV92AaeXatLxBI9gBaebbnrfifHhDYfgasaacH8akY=wiFfYdH8Gipec8Eeeu0xXdbba9frFj0=OqFfea0dXdd9vqai=hGuQ8kuc9pgc9s8qqaq=dirpe0xb9q8qiLsFr0=vr0=vr0dc8meaabaqaciaacaGaaeqabaqabeGadaaakeaacyGGSbaBcqGGVbWBcqGGNbWzcqGGOaakcqaHbpGCdaWgaaWcbaGaee4AaSMaeeiAaGgabeaakiabcMcaPiabg2da9iabeg7aHnaaBaaaleaacqqGRbWAaeqaaOGaey4kaSIaeq4TdG2aa0baaSqaaiabbUgaRjabbMgaPnaaBaaameaacqqGObaAaeqaaaWcbaGaeiikaGIaeeyyaeMaeiykaKcaaOGaey4kaSIaeq4TdG2aa0baaSqaaiabbUgaRjabbQgaQnaaBaaameaacqqGObaAaeqaaaWcbaGaeiikaGIaeeiCaaNaeiykaKcaaOGaey4kaSIaeeyDau3aaSbaaSqaaiabbUgaRjabbIgaObqabaGccaWLjaGaaCzcamaabmaabaGaeeyrauKaeeyCaeNaeeyDauNaeeyyaeMaeeiDaqNaeeyAaKMaee4Ba8MaeeOBa4MaeeiiaaIaeGymaeJaeGymaedacaGLOaGaayzkaaaaaa@64FD@

where (u_1h_, u_2h_,..u_Kh_) ~ N_K_(0, Σ_u_). It may be that only one outcome (say k*) is not satisfactorily predicted and in this case univariate unstructured effects u_k*h _at cell level may be introduced for that outcome only. Another possibility for model extension is heterogeneity over SOAs in the effects of known predictors in equation (8), such as varying impacts of GP access or distance to hospital. Such heterogeneity can itself be modelled using an MCAR prior over outcomes k.

Multivariate correlation in errors does not necessarily require a conventional K dimension error density (e.g. a K-variate normal). One possible alternative approach assumes that the s_ki _and the e_kj _are generated by a single area factor s_i _and a single practice factor e_j _respectively, with outcome specific loadings governing the relative importance of the common factor to explaining each outcome. Under a single common factor model the area and practice components have the form

η^(a)^_ki _= γ_k1_AHN_i _+ γ_k2_A_i _+ γ_k3_R_i _+ κ_k_s_i _    (Equation 12)

η^(p)^_kj _= β_k1_PHN_j _+ β_k2_NH_j _+ λ_k_e_j _    (Equation 13)

where {κ_k_, λ_k_} are loadings. Whether all the event specific loadings κ_k _and λ_k _are free parameters depends on whether the variances σs2
 MathType@MTEF@5@5@+=feaafiart1ev1aaatCvAUfKttLearuWrP9MDH5MBPbIqV92AaeXatLxBI9gBaebbnrfifHhDYfgasaacH8akY=wiFfYdH8Gipec8Eeeu0xXdbba9frFj0=OqFfea0dXdd9vqai=hGuQ8kuc9pgc9s8qqaq=dirpe0xb9q8qiLsFr0=vr0=vr0dc8meaabaqaciaacaGaaeqabaqabeGadaaakeaacqaHdpWCdaqhaaWcbaGaee4CamhabaGaeGOmaidaaaaa@30FB@ and σe2
 MathType@MTEF@5@5@+=feaafiart1ev1aaatCvAUfKttLearuWrP9MDH5MBPbIqV92AaeXatLxBI9gBaebbnrfifHhDYfgasaacH8akY=wiFfYdH8Gipec8Eeeu0xXdbba9frFj0=OqFfea0dXdd9vqai=hGuQ8kuc9pgc9s8qqaq=dirpe0xb9q8qiLsFr0=vr0=vr0dc8meaabaqaciaacaGaaeqabaqabeGadaaakeaacqaHdpWCdaqhaaWcbaGaeeyzaugabaGaeGOmaidaaaaa@30DF@ of s_i _and e_j _are preset [41].

### Policy Relevant Model Outputs

Measures to reduce high attendance rates may involve provision of extra primary care clinics or staff and the siting of such resources is important. A possible index of poor access in relation to health care need is the discrepancy between the attendance or admission relative risks in different areas (μ_ki_) and levels of access to primary care. The latter is measured by the ratio of access in SOA i to average access in all SOAs, namely A_i_/A¯
 MathType@MTEF@5@5@+=feaafiart1ev1aaatCvAUfKttLearuWrP9MDH5MBPbIqV92AaeXatLxBI9gBaebbnrfifHhDYfgasaacH8akY=wiFfYdH8Gipec8Eeeu0xXdbba9frFj0=OqFfea0dXdd9vqai=hGuQ8kuc9pgc9s8qqaq=dirpe0xb9q8qiLsFr0=vr0=vr0dc8meaabaqaciaacaGaaeqabaqabeGadaaakeaacuqGbbqqgaqeaaaa@2DCD@. So

Δ_ki _= μ_ki_-A_i_/A¯
 MathType@MTEF@5@5@+=feaafiart1ev1aaatCvAUfKttLearuWrP9MDH5MBPbIqV92AaeXatLxBI9gBaebbnrfifHhDYfgasaacH8akY=wiFfYdH8Gipec8Eeeu0xXdbba9frFj0=OqFfea0dXdd9vqai=hGuQ8kuc9pgc9s8qqaq=dirpe0xb9q8qiLsFr0=vr0=vr0dc8meaabaqaciaacaGaaeqabaqabeGadaaakeaacuqGbbqqgaqeaaaa@2DCD@     (Equation 14)

is a measure suggesting where demand for A&E attendances or admissions might be reduced by improving access to primary care. From equations 3 and 4, A_i _is stochastic so the Δ_ki _need to be monitored during the MCMC sampling and a posterior density obtained. High posterior means in Δ_ki _(or in the ranks of Δ_ki_) in particular SOAs will occur when a high attendance or admission rate is combined with access below average.

Also relevant are measures of practice referral behaviour after controlling for known influences on health need (i.e. controlling for the level of morbidity of the population that a GP practice serves). These are represented here by the residuals e_kj _under the multivariate error models, or the pooled e_j _under the common factor model. Posterior densities of ranks on such residuals are useful in assessing performance in terms of referral rates in excess of health need, and have been used in other institutional comparisons [42,43].

### Model Estimation and Priors

The analysis starts by comparing two models, the first based on fixed effects only using the observed predictors. Thus for the fixed effects model

η^(a)^_ki _= γ_k1_AHN_i _+ γ_k2_A_i _+ γ_k3_R_i _    (Equation 15)

η^(p)^_kj _= β_k1_PHN_j _+ β_k2_NH_j_.

The second model assumes a full dimension error structure for both practice and SOA effects but has no cell level effects. Relatively diffuse N(0,1000) priors are used for the fixed effects {α_k_, β_k1_, β_k2_, γ_k1_, γ_k2_, γ_k3_} under all models. For the full dimension error models, Wishart priors with identity scale matrix and K = 2 degrees of freedom are assumed for the precision matrices Σs−1
 MathType@MTEF@5@5@+=feaafiart1ev1aaatCvAUfKttLearuWrP9MDH5MBPbIqV92AaeXatLxBI9gBaebbnrfifHhDYfgasaacH8akY=wiFfYdH8Gipec8Eeeu0xXdbba9frFj0=OqFfea0dXdd9vqai=hGuQ8kuc9pgc9s8qqaq=dirpe0xb9q8qiLsFr0=vr0=vr0dc8meaabaqaciaacaGaaeqabaqabeGadaaakeaacqqHJoWudaqhaaWcbaGaee4CamhabaGaeyOeI0IaeGymaedaaaaa@31A7@ and Σe−1
 MathType@MTEF@5@5@+=feaafiart1ev1aaatCvAUfKttLearuWrP9MDH5MBPbIqV92AaeXatLxBI9gBaebbnrfifHhDYfgasaacH8akY=wiFfYdH8Gipec8Eeeu0xXdbba9frFj0=OqFfea0dXdd9vqai=hGuQ8kuc9pgc9s8qqaq=dirpe0xb9q8qiLsFr0=vr0=vr0dc8meaabaqaciaacaGaaeqabaqabeGadaaakeaacqqHJoWudaqhaaWcbaGaeeyzaugabaGaeyOeI0IaeGymaedaaaaa@318B@.

The remaining unknown in both models is the distance decay parameter in the exponential model for GP access scores in equation (4). To model b as a continuous unknown would be at the cost of considerably greater computing times, because the need for repeated calculation of f(d_ij_) involves a 149 × 53 distance matrix. So a 24 point discrete prior for b on values {0.1,0.15,0.2,0.25,0.3,...,0.9,0.95,1,1.1,1.2,1.3,1.4,1.5} is assumed using pre-calculated decay matrices at these values. This range of values accords with prior knowledge; for example, Carr-Hill et al [36] assume b = 0.2.

Fit is measured using the Deviance Information Criterion (abbreviated DIC) of Spiegelhalter et al [44], considered in terms of the separate outcomes. Denote the posterior mean of the deviance as D¯
 MathType@MTEF@5@5@+=feaafiart1ev1aaatCvAUfKttLearuWrP9MDH5MBPbIqV92AaeXatLxBI9gBaebbnrfifHhDYfgasaacH8akY=wiFfYdH8Gipec8Eeeu0xXdbba9frFj0=OqFfea0dXdd9vqai=hGuQ8kuc9pgc9s8qqaq=dirpe0xb9q8qiLsFr0=vr0=vr0dc8meaabaqaciaacaGaaeqabaqabeGadaaakeaacuqGebargaqeaaaa@2DD3@_k _= -2L¯
 MathType@MTEF@5@5@+=feaafiart1ev1aaatCvAUfKttLearuWrP9MDH5MBPbIqV92AaeXatLxBI9gBaebbnrfifHhDYfgasaacH8akY=wiFfYdH8Gipec8Eeeu0xXdbba9frFj0=OqFfea0dXdd9vqai=hGuQ8kuc9pgc9s8qqaq=dirpe0xb9q8qiLsFr0=vr0=vr0dc8meaabaqaciaacaGaaeqabaqabeGadaaakeaacuqGmbatgaqeaaaa@2DE3@_k _where L_k _is the log-likelihood for event k taken over all cells h. The DIC for outcome k is D¯
 MathType@MTEF@5@5@+=feaafiart1ev1aaatCvAUfKttLearuWrP9MDH5MBPbIqV92AaeXatLxBI9gBaebbnrfifHhDYfgasaacH8akY=wiFfYdH8Gipec8Eeeu0xXdbba9frFj0=OqFfea0dXdd9vqai=hGuQ8kuc9pgc9s8qqaq=dirpe0xb9q8qiLsFr0=vr0=vr0dc8meaabaqaciaacaGaaeqabaqabeGadaaakeaacuqGebargaqeaaaa@2DD3@_k _plus a complexity penalty c_k_, estimated as c_k _= D¯
 MathType@MTEF@5@5@+=feaafiart1ev1aaatCvAUfKttLearuWrP9MDH5MBPbIqV92AaeXatLxBI9gBaebbnrfifHhDYfgasaacH8akY=wiFfYdH8Gipec8Eeeu0xXdbba9frFj0=OqFfea0dXdd9vqai=hGuQ8kuc9pgc9s8qqaq=dirpe0xb9q8qiLsFr0=vr0=vr0dc8meaabaqaciaacaGaaeqabaqabeGadaaakeaacuqGebargaqeaaaa@2DD3@_k _- D(θ¯
 MathType@MTEF@5@5@+=feaafiart1ev1aaatCvAUfKttLearuWrP9MDH5MBPbIqV92AaeXatLxBI9gBaebbnrfifHhDYfgasaacH8akY=wiFfYdH8Gipec8Eeeu0xXdbba9frFj0=OqFfea0dXdd9vqai=hGuQ8kuc9pgc9s8qqaq=dirpe0xb9q8qiLsFr0=vr0=vr0dc8meaabaqaciaacaGaaeqabaqabeGadaaakeaacuaH4oqCgaqeaaaa@2E7A@_k_), where D(θ¯
 MathType@MTEF@5@5@+=feaafiart1ev1aaatCvAUfKttLearuWrP9MDH5MBPbIqV92AaeXatLxBI9gBaebbnrfifHhDYfgasaacH8akY=wiFfYdH8Gipec8Eeeu0xXdbba9frFj0=OqFfea0dXdd9vqai=hGuQ8kuc9pgc9s8qqaq=dirpe0xb9q8qiLsFr0=vr0=vr0dc8meaabaqaciaacaGaaeqabaqabeGadaaakeaacuaH4oqCgaqeaaaa@2E7A@_k_) is the deviance at the mean of the parameter set θ_k _for model k. The extent of overdispersion is assessed by obtaining the posterior mean of the scaled deviance.

The ability of the model to reproduce the data is assessed via predictive checks from the posterior predictive density P(Y_new_|Y), where model predictions (replicate data sampled from the model)

Ynew,kh|θk(t)∼Po(Ekhρkh(t))     (Equation 16)
 MathType@MTEF@5@5@+=feaafiart1ev1aaatCvAUfKttLearuWrP9MDH5MBPbIqV92AaeXatLxBI9gBaebbnrfifHhDYfgasaacH8akY=wiFfYdH8Gipec8Eeeu0xXdbba9frFj0=OqFfea0dXdd9vqai=hGuQ8kuc9pgc9s8qqaq=dirpe0xb9q8qiLsFr0=vr0=vr0dc8meaabaqaciaacaGaaeqabaqabeGadaaakeaacqqGzbqwdaWgaaWcbaGaeeOBa4MaeeyzauMaee4DaCNaeiilaWIaee4AaSMaeeiAaGgabeaakiabcYha8jabeI7aXnaaDaaaleaacqqGRbWAaeaacqGGOaakcqqG0baDcqGGPaqkaaGccqWI8iIocqqGqbaucqqGVbWBcqGGOaakcqqGfbqrdaWgaaWcbaGaee4AaSMaeeiAaGgabeaakiabeg8aYnaaDaaaleaacqqGRbWAcqqGObaAaeaacqGGOaakcqqG0baDcqGGPaqkaaGccqGGPaqkcaWLjaGaaCzcamaabmaabaGaeeyrauKaeeyCaeNaeeyDauNaeeyyaeMaeeiDaqNaeeyAaKMaee4Ba8MaeeOBa4MaeeiiaaIaeGymaeJaeGOnaydacaGLOaGaayzkaaaaaa@5F33@

are sampled at iterations t = T_1_,..T_2 _from the MCMC sample chains, and iterations 1,..T_1 _constitute the convergence stage. Following Gelfand [45], a check is made whether the observed Y_kh _are within 95% intervals of the predictions Y_new,kh_. For a satisfactory model the 95% intervals of the predictions should include the actual observations with probability of 0.95 or higher.

Estimation uses two chains with dispersed initial values run for 10000 iterations. Convergence was assessed by Gelman-Rubin criteria [46], and summaries of the parameters, the discrepancy indices Δ_ki_, and GP practice error ranks, are based on iterations 5,000–10,000.

## Results

Table 3 shows that a random errors interdependent outcomes model (model 2) has better fit than the fixed effects model 1 as measured by the DIC, albeit at the cost of an increase in complexity. The fixed effects model shows overdispersion in relation to the total number of observations (namely 1610 for each outcome and 3220 for both outcomes combined). The random effects model provides satisfactory predictions of the data for attendances, and removes overdispersion in attendances. However, some predictive discrepancies remain for admissions, since only 91.4% of the 95% credible intervals for replicate data include the actual data. The scaled deviance for admissions under model 2 still indicates overdispersion.

**Table 3:** Model Fit and Checking Criteria

	**Model 1**					**Model 2**					**Model 3**				
	
	**Fixed Effects Only**					**Full Dimension SOA & Practice errors**					**Full Dimension SOA & Practice errors, Cell errors for admissions**				
	
	**Mean Deviances**	**Scaled Deviances (Posterior Mean)**	**c_k_**	**DIC**	**% of y_h _in 95% intervals of y_rep,h_**	**Mean Deviances**	**Scaled Deviances (Posterior Mean)**	**c_k_**	**DIC**	**% of y_h _in 95% intervals of y_rep,h_**	**Mean Deviances**	**Scaled Deviances (Posterior Mean)**	**c_k_**	**DIC**	**% of y_h _in 95% intervals of y_rep,h_**
Attendances	7992	2054	6.4	7998	93.6	6192	1492	114	6306	100.0	6166	1405	135	6301	100.0
Admissions	6707	3489	3.9	6711	88.0	5966	2805	116	6082	92.1	5005	1792	596	5601	98.9
Both Events	14699	5543	10.3	14709	90.8	12158	4297	230	12388	96.0	11171	3197	731	11902	99.4

Coefficient summaries (Table 4) for these two models show that allowing for unobserved area effects (e.g. unobserved influences on morbidity and access) enhances the distance effect to hospital parameters, γ_k3_. Such enhancements of fixed effect parameters (in absolute terms) are often obtained in random effects models. The coefficients γ_k1 _and β_k1 _reflecting the impact of composite health need are both significant, and enhanced in model 2. The impacts of access to primary care (increases in which might be expected to reduce emergency admissions and attendances), as summarised in the parameter γ_k2_, are negative. In both models the posterior densities for b_k _in f_k_(d_ij_) concentrate on small values.

**Table 4:** Parameter Estimates

**Outcome and Predictor**		**Model 1**		**Model 2**		**Model 3**	
		
		**Fixed Effects Only**		**Full Dimension area & practice errors**		**Full Dimension area & practice errors; cell level admission errors**	
Attendances		Mean	s.d.	Mean	s.d.	Mean	s.d.

γ_11_	Area Need	0.08	0.01	0.10	0.02	0.09	0.01
γ_12_	Primary Care Access	-0.24	0.06	-0.68	0.08	-0.92	0.11
γ_13_	Distance to Hospital	-0.04	0.005	-0.22	0.02	-0.22	0.02
β_11_	Practice Need	0.12	0.01	0.27	0.04	0.24	0.03
β_12_	Nursing Home Patients	0.29	0.06	0.67	0.22	0.61	0.23
Admissions							
γ_21_	Area Need	0.06	0.02	0.10	0.04	0.08	0.03
γ_22_	Primary Care Access	-0.66	0.07	-1.01	0.09	-0.71	0.14
γ_23_	Distance to Hospital	-0.09	0.01	-0.38	0.04	-0.27	0.03
β_21_	Practice Need	0.12	0.02	0.28	0.07	0.34	0.05
β_22_	Nursing Home Patients	0.39	0.10	0.83	0.27	1.06	0.13
Distance Decay Parameters, b_k_							
Attendances		0.16	0.08	0.36	0.08	0.27	0.09
Admissions		0.25	0.06	0.49	0.09	0.45	0.11

A third model seeks to improve fit and predictions for admissions by adding a cell level random error for k = 2. So

log⁡(ρ1h)=α1+η1ih(a)+η1jh(p)     (Equation 17)log⁡(ρ2h)=α2+η2ih(a)+η2jh(p)+u2h
 MathType@MTEF@5@5@+=feaafiart1ev1aaatCvAUfKttLearuWrP9MDH5MBPbIqV92AaeXatLxBI9gBaebbnrfifHhDYfgasaacH8akY=wiFfYdH8Gipec8Eeeu0xXdbba9frFj0=OqFfea0dXdd9vqai=hGuQ8kuc9pgc9s8qqaq=dirpe0xb9q8qiLsFr0=vr0=vr0dc8meaabaqaciaacaGaaeqabaqabeGadaaakeaafaqaaeGabaaabaGagiiBaWMaei4Ba8Maei4zaCMaeiikaGIaeqyWdi3aaSbaaSqaaiabigdaXiabbIgaObqabaGccqGGPaqkcqGH9aqpcqaHXoqydaWgaaWcbaGaeGymaedabeaakiabgUcaRiabeE7aOnaaDaaaleaacqaIXaqmcqqGPbqAdaWgaaadbaGaeeiAaGgabeaaaSqaaiabcIcaOiabbggaHjabcMcaPaaakiabgUcaRiabeE7aOnaaDaaaleaacqaIXaqmcqqGQbGAdaWgaaadbaGaeeiAaGgabeaaaSqaaiabcIcaOiabbchaWjabcMcaPaaakiaaxMaacaWLjaWaaeWaaeaacqqGfbqrcqqGXbqCcqqG1bqDcqqGHbqycqqG0baDcqqGPbqAcqqGVbWBcqqGUbGBcqqGGaaicqaIXaqmcqaI3aWnaiaawIcacaGLPaaaaeaacyGGSbaBcqGGVbWBcqGGNbWzcqGGOaakcqaHbpGCdaWgaaWcbaGaeGOmaiJaeeiAaGgabeaakiabcMcaPiabg2da9iabeg7aHnaaBaaaleaacqaIYaGmaeqaaOGaey4kaSIaeq4TdG2aa0baaSqaaiabikdaYiabbMgaPnaaBaaameaacqqGObaAaeqaaaWcbaGaeiikaGIaeeyyaeMaeiykaKcaaOGaey4kaSIaeq4TdG2aa0baaSqaaiabikdaYiabbQgaQnaaBaaameaacqqGObaAaeqaaaWcbaGaeiikaGIaeeiCaaNaeiykaKcaaOGaey4kaSIaeeyDau3aaSbaaSqaaiabikdaYiabbIgaObqabaaaaaaa@841C@

where u_2h _~ N(0, σu2
 MathType@MTEF@5@5@+=feaafiart1ev1aaatCvAUfKttLearuWrP9MDH5MBPbIqV92AaeXatLxBI9gBaebbnrfifHhDYfgasaacH8akY=wiFfYdH8Gipec8Eeeu0xXdbba9frFj0=OqFfea0dXdd9vqai=hGuQ8kuc9pgc9s8qqaq=dirpe0xb9q8qiLsFr0=vr0=vr0dc8meaabaqaciaacaGaaeqabaqabeGadaaakeaacqaHdpWCdaqhaaWcbaGaeeyDauhabaGaeGOmaidaaaaa@30FF@), and 1/σu2
 MathType@MTEF@5@5@+=feaafiart1ev1aaatCvAUfKttLearuWrP9MDH5MBPbIqV92AaeXatLxBI9gBaebbnrfifHhDYfgasaacH8akY=wiFfYdH8Gipec8Eeeu0xXdbba9frFj0=OqFfea0dXdd9vqai=hGuQ8kuc9pgc9s8qqaq=dirpe0xb9q8qiLsFr0=vr0=vr0dc8meaabaqaciaacaGaaeqabaqabeGadaaakeaacqaHdpWCdaqhaaWcbaGaeeyDauhabaGaeGOmaidaaaaa@30FF@ is assigned a gamma G(1,0.001) prior. Predictive checks for this model are satisfactory, and despite increased complexity for the admissions component (with c_2 _= 596), the DIC for all parts of the model is improved over model 2. The remaining overdispersion in admissions is only slight.

Model 3 is therefore used to make inferences for particular areas or practices that are of interest in health strategy terms. For practices, discrepancies against anticipated utilisation are assessed by the residuals e_kj _in equation (9), which control for known need and access influences on utilisation. Table 5 lists posterior medians and 95% intervals for ranks on residual practice effects e_kj _under model 3 (highest positive residuals have higher ranks), together with practice population needs scores, obtained via factor analysis from four measured indicators. Also provided are estimated practice relative risks ν_kj _by event k.

**Table 5:** Practice Level Outcomes from Area-Practice Model 3

	**ν_1j_, (RR attendances)**		**ν_2j_, (RR admissions)**		**Rank on e_1j_**			**Rank on e_2j_**			**Practice Needs Score**
**Practice**	**Mean**	**St devn**	**Mean**	**St devn**	**2.5%**	**Median**	**97.5%**	**2.5%**	**Median**	**97.5%**	

1	0.85	0.06	1.07	0.12	9	16	24	16	21	29	-1.5
2	0.75	0.05	1.06	0.10	9	18	28	36	41	46	-1.5
3	1.58	0.05	1.28	0.17	48	49	50	23	28	33	-0.1
4	1.04	0.03	1.36	0.11	28	36	40	45	47	49	-0.4
5	0.99	0.04	1.10	0.07	20	27	32	23	31	37	-0.2
6	1.42	0.08	1.53	0.21	9	12	16	10	13	15	2.1
7	1.20	0.05	1.58	0.23	26	34	40	36	41	46	0.5
8	0.94	0.03	1.14	0.10	17	26	33	35	38	43	-0.4
9	1.22	0.07	1.63	0.19	6	6	8	7	11	13	2.1
10	1.54	0.09	1.55	0.21	9	14	24	7	9	15	2.3
11	1.69	0.24	2.86	0.95	50	51	53	51	53	53	0.1
12	1.06	0.06	1.55	0.18	17	24	33	35	42	47	0.2
13	1.25	0.05	1.20	0.10	32	41	46	18	23	27	-0.8
14	1.18	0.04	1.32	0.17	39	45	48	32	34	38	-0.3
15	1.11	0.06	1.18	0.14	23	35	43	38	44	47	0.1
16	1.08	0.05	1.36	0.15	13	16	23	20	27	31	0.5
17	0.29	0.03	0.22	0.07	2	3	3	3	3	4	0.6
18	0.94	0.06	0.97	0.15	29	38	45	29	42	49	-0.9
19	1.78	0.16	1.50	0.35	17	30	43	5	7	12	0.4
20	1.14	0.07	1.56	0.21	19	28	34	30	37	46	0.3
21	1.21	0.24	1.38	0.71	11	36	50	11	36	52	0.1
22	1.10	0.08	0.93	0.17	17	27	38	5	9	15	-1.3
23	0.86	0.07	0.94	0.14	20	29	42	20	40	47	-0.9
24	1.13	0.11	1.08	0.23	38	47	49	44	50	51	-0.3
25	0.83	0.07	0.77	0.15	11	33	42	16	26	45	-1.8
26	1.06	0.08	1.07	0.14	28	39	46	23	31	46	-0.7
27	1.02	0.07	1.04	0.19	26	34	43	21	36	45	0.2
28	1.28	0.09	1.63	0.30	38	45	49	50	50	51	0.2
29	1.37	0.09	1.23	0.18	42	49	50	26	37	46	0.0
30	0.64	0.07	0.88	0.17	4	5	6	6	16	33	0.5
31	0.81	0.09	0.56	0.14	10	16	27	5	9	18	-0.3
32	0.15	0.03	0.04	0.02	1	1	3	1	1	2	-0.4
33	0.74	0.06	1.16	0.18	6	8	12	12	18	25	-0.9
34	1.02	0.09	0.99	0.21	17	28	45	16	21	32	0.3
35	2.16	0.17	3.15	0.49	51	52	53	52	52	53	0.7
36	1.43	0.09	1.94	0.24	8	11	16	8	12	16	0.6
37	0.85	0.08	0.23	0.07	6	10	23	3	4	4	0.4
38	1.31	0.07	1.26	0.15	33	42	48	24	32	38	0.4
39	0.93	0.08	1.03	0.20	5	7	10	5	6	15	-0.5
40	0.83	0.07	0.96	0.17	12	23	36	12	24	34	-1.5
41	0.20	0.03	0.05	0.02	1	2	3	1	2	2	0.6
42	2.12	0.10	1.68	0.19	51	52	53	42	48	50	0.3
43	1.29	0.09	1.04	0.19	8	11	20	5	6	9	2.0
44	1.04	0.09	1.72	0.36	14	20	34	35	43	49	0.0
45	0.96	0.07	0.91	0.18	17	34	42	12	15	21	-0.8
46	0.75	0.07	0.82	0.16	10	17	28	17	27	35	-1.5
47	1.27	0.08	1.43	0.20	37	46	49	41	47	50	0.0
48	0.83	0.07	0.80	0.18	15	22	35	11	19	31	-0.6
49	1.97	0.14	1.82	0.30	25	39	48	13	19	31	2.2
50	1.73	0.11	1.97	0.26	18	32	43	17	23	27	2.0
51	1.00	0.07	1.16	0.16	33	42	49	19	33	43	-1.4
52	0.90	0.05	1.02	0.12	9	16	24	22	29	33	-0.8
53	0.53	0.06	0.98	0.22	4	4	5	9	17	30	0.4

There is a correlation of 0.74 between the posterior ranks on e_1j _and those on e_2j_, demonstrating overlapping issues of GP practice referral behaviour for the two outcomes. There is overlap between the results in Table 5 and the earlier reported analysis based on maximum likelihood SARs and classical 95% intervals. However, practices 36 and 50 no longer figure as leading admission rate outliers (in terms of median ranks on residuals e_k2_) after taking account of their need scores and nursing home patients; similarly, practices 19 and 49 no longer figure as attendance rate outliers.

**Figure 4:**
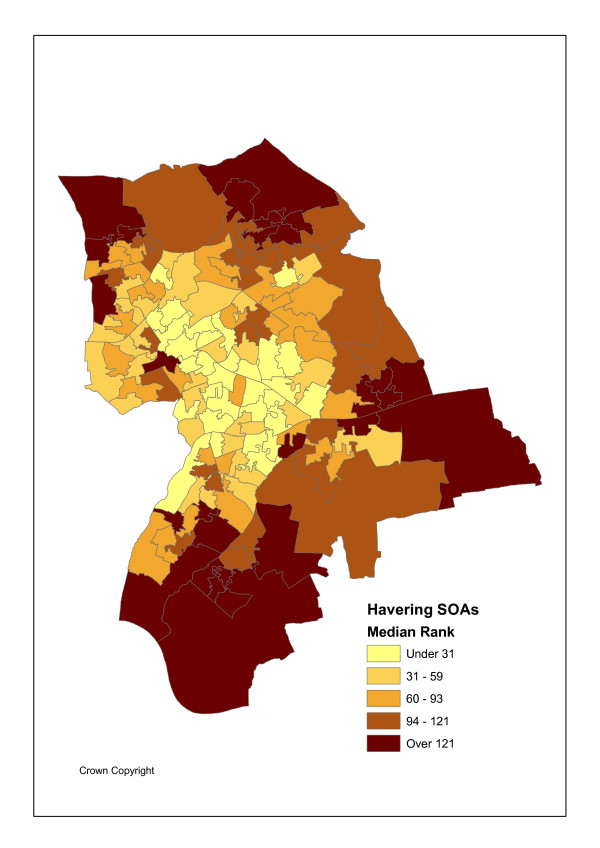
Ranks on Attendance Residuals.

**Figure 5:**
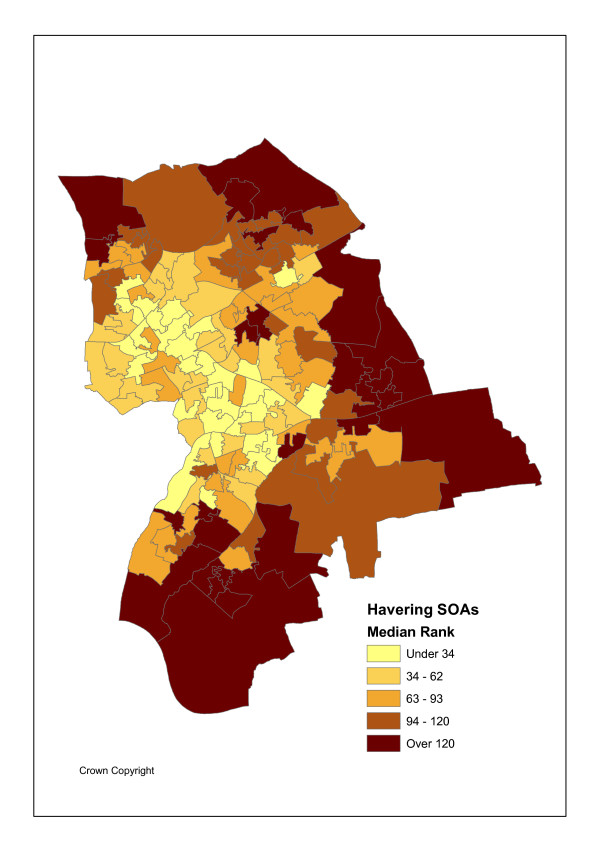
Ranks on Admission Residuals.

Outlier areas are also important to identify when excess hospital attendances or admissions by small area coincide with relatively poor access to primary care. Figures 4 and 5 shows the distribution of the median posterior ranks of the discrepancies in equation (14). These two sets of discrepancies are highly related (a correlation of 0.94 between the 149 posterior medians for the two outcomes). There are also positive correlations between such discrepancies (as measured by posterior median ranks) and the event relative risks themselves (0.62 for attendances, and 0.59 for admissions). There are also positive correlations between the discrepancies in equation (14) and area IMD scores (0.49 for attendances, and 0.30 for admissions), demonstrating an interaction between relatively low primary care access and social deprivation in causing potentially avoidable hospital utilisation.

## Discussion

Modern health care strategy emphasises the need for avoiding unnecessary referrals to acute care and the need for effective health care strategies for long term chronic conditions that are based in primary and community care settings. The paper has sought to present a methodology that analyses interactions between population casemix and the referral behaviour of primary care "gatekeepers" and that also reflects interdependence between outcomes. Such a methodology can be widely applied as ensuring an effective and appropriate balance between primary and secondary care in the management of chronic disease is a recurrent issue. Particular goals might include identifying whether there are areas or GP practices with significantly elevated referral rates, especially when not justified by morbidity.

The analysis of the paper has had a specialised focus on a relatively affluent outer city area and on two particular outcomes, namely emergency admissions and attendances at hospitals. Such forms of hospital use are often assessed as unnecessary, especially when involving conditions that could be treated in primary care settings. The present analysis identified GP practices that had high attendance and admission rates in relation to the needs level in their catchment populations (e.g. practices 11, 35 and 42 in Table 5), and this is also apparent in median ranks on residuals after controlling for need. While admitting that health care need may often have multiple separate dimensions, in the case study a selection of commonly used indicators seem to be measuring essentially the same dimension (Table 2) and a factor analysis was used to combine the multiple measured needs indices.

The present study has considered multiple interrelated outcomes but only for a single hospital. For many populations (e.g. in rural areas, relatively small towns, or outer suburban areas as in the case study) this reflects a reality of a single effective provider except for less common elective procedures where 'out of area' referrals are possible. However, many urbanised inner city areas have a choice of providers (e.g. general hospitals with emergency units). If observations Y_khm _are available by area-practice cell h, outcome k, and relevant providers m = 1,..M, then the model can be provider specific, with Y_khm _~ Po(E_kh_ρ_khm_). This model would include supply factors (e.g. bed/staff numbers) in an additional provider/facility term η^(f)^_km_, as well as allowing for residence-hospital interactions η^(a)^_kim_. So for i = i_h _and j = j_h_

log⁡(ρkhm)=αkm+ηkihm(a)+ηkjh(p)+ηkm(f).     (Equation 18)
 MathType@MTEF@5@5@+=feaafiart1ev1aaatCvAUfKttLearuWrP9MDH5MBPbIqV92AaeXatLxBI9gBaebbnrfifHhDYfgasaacH8akY=wiFfYdH8Gipec8Eeeu0xXdbba9frFj0=OqFfea0dXdd9vqai=hGuQ8kuc9pgc9s8qqaq=dirpe0xb9q8qiLsFr0=vr0=vr0dc8meaabaqaciaacaGaaeqabaqabeGadaaakeaacyGGSbaBcqGGVbWBcqGGNbWzcqGGOaakcqaHbpGCdaWgaaWcbaGaee4AaSMaeeiAaGMaeeyBa0gabeaakiabcMcaPiabg2da9iabeg7aHnaaBaaaleaacqqGRbWAcqqGTbqBaeqaaOGaey4kaSIaeq4TdG2aa0baaSqaaiabbUgaRjabbMgaPnaaBaaameaacqqGObaAaeqaaSGaeeyBa0gabaGaeiikaGIaeeyyaeMaeiykaKcaaOGaey4kaSIaeq4TdG2aa0baaSqaaiabbUgaRjabbQgaQnaaBaaameaacqqGObaAaeqaaaWcbaGaeiikaGIaeeiCaaNaeiykaKcaaOGaey4kaSIaeq4TdG2aa0baaSqaaiabbUgaRjabb2gaTbqaaiabcIcaOiabbAgaMjabcMcaPaaakiabc6caUiaaxMaacaWLjaWaaeWaaeaacqqGfbqrcqqGXbqCcqqG1bqDcqqGHbqycqqG0baDcqqGPbqAcqqGVbWBcqqGUbGBcqqGGaaicqaIXaqmcqaI4aaoaiaawIcacaGLPaaaaaa@6D5D@

In the origin-hospital interaction component, distances to hospital (R_im_) would be specific to each area and provider, so that for i = i_h_

η^(a)^_kim _= γ_k1_AHN_i _+ γ_k2_A_i _+ γ_k3_R_im _+ s_ki_.     (Equation 19)

Alternatively, an access to hospital term (allowing for both hospital mass and distances from areas to hospitals) could replace R_im _in equation 19.

The present study has considered particular relatively broad outcomes and a particular geographic setting. Different outcomes, specific to particular diagnostic or disease groups, or to different types of geographic setting, would need to adapt the model. For instance, compared to other London boroughs, Havering is relatively affluent, with lower illness and deprivation levels than average. However, it is internally diverse, containing some deprived pockets of social rented housing. In other London boroughs features such as ethnic mix may play a greater role in explaining some types of referral activity [47]. Havering is a largely affluent and white borough (around 95% white in 2001, the highest in London), with some resemblance to less metropolitan areas outside London, in having pockets of deprivation, and in having low homeless and refugee populations. Analysis of particular outcomes in different geographic settings, such as cardiovascular or mental illness referrals in inner city areas, would need to include ethnicity, as well as the significant presence of marginalised groups, as influences on need.
